# Secure healthcare data management using federated learning, blockchain, and explainable artificial intelligence: a systematic review

**DOI:** 10.3389/fdgth.2026.1871960

**Published:** 2026-06-03

**Authors:** Tanisha Bhardwaj, K. Sumangali

**Affiliations:** School of Computer Science Engineering and Information Systems, Vellore Institute of Technology, Vellore, India

**Keywords:** blockchain technology, differential privacy, explainable artificial intelligence, federated learning, GDPR compliance, healthcare data security, incremental learning, internet of medical things (IoMT)

## Abstract

Due to the rapid digitization of healthcare systems, there has been a huge collection of sensitive personal data of patients. Thus, secure, privacy-preserving, and efficient data management systems are required. Current distributed healthcare systems increasingly use centralized data processing frameworks that are prone to privacy violations, data fragmentation, and malicious attacks. Despite advances in federated learning, blockchain, explainable AI, and incremental optimization, current survey literature studies each technology separately without considering how the four technologies can be harnessed to create synergies. A systematic review of 26 peer-reviewed studies published from 2018 to 2026 indicates that an integrated architecture incorporating federated learning, blockchain, explainable AI, and incremental optimization can be designed. This review identifies ten critical issues that need to be addressed when researching the four technologies. These issues include communication costs, scalability issues, interoperability concerns, limited clinical explainability, and high computational costs when applied in real-time situations. In comparison to privacy, scalability, interpretability, and efficiency, a hybrid approach can help improve data security, boost the interpretability of the models, facilitate data sharing, and prevent data-sharing risks. Overall quality assessment based on the CASP qualitative checklist analysis of all 26 studies indicated an average score of 7.0 out of 10, implying that the quality of the methods used in the studies was acceptable.

## 1 Introduction

Big data analysis and artificial intelligence are developing rapidly, and the incorporation of medical technology in the healthcare industry is undergoing a large-scale transition (1). The emergence of Electronic Health Records (EHRs) (1), telemedicine programs (3), and IoMT devices (4) has allowed the generation of huge, heterogeneous, and high-dimensional health data (2). This information is useful for diagnosing a disease, determining its progression, and treating it (5). Having sensitive patient data stored and processed centrally raises serious concerns around privacy, data security, and compliance with regulations such as HIPAA (12) and GDPR (11). According to conventional ML methods, data from diverse sources are pooled into a common database. This pooling of data faces threats and risks of cyberattacks, data theft, and unauthorized access (6). Moreover, healthcare organizations often hesitate to share patient data for ethical, regulatory, or competitive reasons, further creating data silos and hampering the effectiveness of AI models. Federated Learning (FL) (7, 8) is a non-centralized learning framework that does not send any data to each other, making collaboration possible among institutions for model training. Federated learning models, called local models, are trained using private data. Only parameters or gradients were transmitted for aggregation. Federated learning might diminish privacy issues; however, it does not guarantee trust, integrity, or security of data and communications.

Enhanced distributed systems can gain insights from blockchain (9) with its architecture of decentralization and immutability, which provides trust, transparency, and security (7). The algorithms and consensus that we use make all transactions unchangeable, auditable and secure. These qualities are particularly valuable in the medical field. As deep learning models become more complex, the explanations for their predictions become increasingly important (8). Using Explainable AI (XAI) techniques (10) enables interpretable and transparent clinical predictions. Healthcare professionals need AI rationale in high-stakes clinical situations to ensure reliability, accountability, and compliance with the law.

In addition, incremental optimization plays a vital role in smart healthcare systems, although it is often ignored. Incremental Optimization allows users to continuously improve models with newly available data without the need for complete retraining, unlike batch learning or fixed optimization (10). In the medical industry, this capability is exceptionally important. As patients' health changes, new diseases are found, and healthcare procedures change, the distribution of the data changes. Owing to Incremental Optimization, convergence can be improved in Federated Learning. This technology will also aid in reducing the computational complexity by enabling instantaneous updates. Furthermore, it boosts the performance of blockchain-integrated systems and dynamic trust-aware updates. Finally, it enhances XAI frameworks by ensuring consistent interpretability over changing models. Consequently, it helps achieve scalable, responsive, and resource-efficient healthcare intelligence.

To develop a secure, scalable, and intelligent healthcare system, this study aims to provide a thorough synthesis of Federated Learning, Blockchain, Explainable AI, and Incremental Optimization.

The following research questions (RQs) serve as an approach for this investigation:

- RQ1: How can Federated Learning safeguard privacy in healthcare data-sharing contexts?
- RQ2: In what ways does blockchain technology augment trust, security, and data integrity in federated healthcare systems?
- RQ3: In what manner does XAI enhance the interpretability and clinical dependability of AI-driven healthcare decisions?
- RQ4: In what manner can Incremental Optimization facilitate ongoing model adaptation and enhance efficiency in dynamic healthcare environments?

The research questions create an orderly analytical framework for the survey, facilitating an analysis of the complementary functions of several technologies and creating a clear link between the introduction, analysis, and conclusions of the article.

### 1.1 Comparison with existing surveys

Several studies have examined subsets of the technologies considered in this study. Existing literature reviews focus on individual technologies, preventing an integrated evaluation of their combined capabilities. In contrast, this study provides an integrated and systematic analysis of Federated Learning, blockchain, Explainable AI, and incremental optimization as the four mutually dependent building blocks for developing an integrated solution for handling healthcare data (54). The relevant surveys are listed in Table 1.

**Table 1:** Comparison of this survey with existing related surveys.

Author/Year	Focus	FL	BC	XAI	Incr. Opt.	Gap vs. This Paper
Madathil et al. (2025) (8)	FL in Healthcare	✓	✗	✗	✗	No BC/XAI/Incremental Learning
Ngoupayou et al. (2025) (39)	FL + Blockchain	✓	✓	✗	✗	No XAI or Incremental Optimization
Sadeghi et al. (2024) (40)	XAI in Healthcare	✗	✗	✓	✗	No FL or Blockchain integration
Shahsavari et al. (2024) (54)	FL + Blockchain	✓	✓	✗	✗	No XAI; no adaptive learning
Hiwale et al. (2023) (45)	BC + FL Telemedicine	✓	✓	✗	✗	No XAI; no incremental methods
This Survey (2026)	FL + BC + XAI + Incr. Opt.	✓	✓	✓	✓	Unified framework—all four combined

Unlike existing surveys that primarily focus on isolated technologies or limited hybrid combinations, this review presents a unified framework integrating Federated Learning, blockchain, Explainable AI, and incremental optimization for secure healthcare data management. Federated Learning enables decentralized privacy-preserving model training, blockchain ensures immutable trust management and secure data sharing, Explainable AI improves transparency and clinical interpretability, and Incremental Optimization supports adaptive learning in continuously evolving healthcare environments. The operational interaction among these technologies establishes a scalable and intelligent ecosystem capable of supporting secure, explainable, and real-time healthcare analysis. Operationally, the four technologies function as a sequential pipeline: Federated Learning trains local models on private institutional data and transmits only model parameters for aggregation; blockchain validates and immutably records these updates through consensus-based smart contracts; Explainable AI generates interpretable rationales for the aggregated model's clinical predictions; and Incremental Optimization enables the model to adapt continuously to new patient data without requiring complete retraining.

As shown in Table 1, existing surveys are either on FL (or blockchain) or some limited combination. The uniqueness of this survey lies in its integration of all four pillars, namely FL, Blockchain, XAI, and Incremental Optimization, into a single cohesive review, thereby filling a significant gap.

## 2 Challenges in secure healthcare data management

In the digital era, health informatics faces one of the greatest challenges in securing health data: as the usage of telemedicine platforms, IoMT devices, and electronic health records continues to rise, the complexity of protecting healthcare data is expected to grow. It is challenging, both technically and organizationally, to ensure compliance with rules like GDPR (11) and HIPAA (12), while ensuring data availability, confidentiality, and integrity. This section outlines the ten core challenges addressed in this review.

- **Privacy leakage in federated learning:** Although federated learning is designed to be decentralized, it can still leak privacy. Adversaries can reconstruct sensitive patient information using gradient inversion attacks. Inference attacks on models can leak patient privacy, as they may reveal whether a data specimen was used during training. The main challenge is that the shared model updates should not leak information of interest while at the same time ensuring model quality.
- **Blockchain Scalability and Latency:** Blockchains that utilize a proof-of-work mechanism do not have good scalability, as the latency is high and throughput is low. In realistic health service scenarios, it is essential to process data quickly and efficiently.
- **Communication Overhead:** Federated Learning requires regular communication between users and a central server to construct a model. In major healthcare rollouts, this can absorb a large amount of bandwidth and induce latencies that can disrupt real-time clinical activity.
- **Data Heterogeneity and Non-IID Distribution:** Healthcare data from various institutions can vary significantly in format, quality, and statistical distribution. The characteristics of such data, which are non-IID, can slow convergence and reduce the generalizability of federated models.
- **Lack of Interpretability:** Deep learning models for healthcare are often seen as “black boxes” that never provide an explanation for the decision-making process. This inability to interpret restricts clinical implementation because health professionals require rationally explainable and transparent predictions.
- **Integration Complexity:** Combining FL, Blockchain, XAI, and optimization in one model leads to strong architectural complexity, whose adjustments and computational costs must be carefully handled.
- **Resource Constraints:** The Internet of Medical Things and edge devices are utility devices that allow data collection and processing, but they are resource-constrained. These limitations make it difficult to implement machine learning models and blockchain systems on these devices.
- **Security Risks:** The Decentralized healthcare systems are susceptible to model poisoning attacks in FL, Sybil attacks in blockchain networks, and threats of security breaches for unauthorized access, which will hamper data integrity and confidentiality.
- **Regulatory Compliance:** Healthcare regulations, like GDPR and HIPAA, impose strict obligations on data-sharing, storing and processing. Thus, it poses a challenge to the adoption of decentralized architectures because data will flow across borders and jurisdictions.
- **Incremental Learning Challenges:** Continuous adapting systems often face difficulties associated with catastrophic forgetting, model drift, and noise accumulation over time. The influence of dynamic healthcare situations could reduce the long-term predictive performance.

## 3 Research methodology

This section describes the systematic methodology applied to identify, screen, and evaluate literature on secure healthcare data management. To ensure methodological transparency and reproducibility, the literature selection process followed the PRISMA-based systematic review protocol. Articles were identified using predefined Boolean search combinations across the IEEE Xplore, ACM Digital Library, Scopus, Web of Science, and ScienceDirect databases. The search process emphasized studies related to Federated Learning, blockchain, Explainable AI, privacy-preserving healthcare systems, and incremental optimization. Duplicate articles were removed before screening, and each study was evaluated independently using the inclusion and exclusion criteria. The final study selection was confirmed through full-text assessment and quality appraisal using the CASP checklist.

### 3.1 Literature search and data collection

Five main databases, namely IEEE Xplore, ACM Digital Library, Scopus, Web of Science, and ScienceDirect, were used to conduct the literature search. The following keywords and Boolean combinations were used in the search: “Secure Healthcare Data Management,” “Blockchain in Healthcare,” “Federated Learning,” “Explainable AI in Medicine,” and “Privacy Preservation Techniques.” Only peer-reviewed papers published between 2018 and 2026 were searched to ensure that the research was based on foundational and recent work. Systematic searching was performed from January to March 2026 with the use of the following Boolean searches on all five databases: (i) “Federated Learning” AND “healthcare” AND “privacy”; (ii) “blockchain” AND “electronic health records” AND “security”; (iii) “Explainable AI” AND “healthcare”; (iv) “Federated Learning” AND “blockchain” AND “medical”; and (v) (“incremental learning” OR “continual learning”) AND “healthcare”.

### 3.2 Inclusion and exclusion criteria

- **Inclusion Criteria:** Articles and papers from peer-reviewed journals and conference papers published in English between 2018 and 2026; studies that directly address data Security, Blockchain, Federated Learning, XAI, and privacy preservation; and articles available in full text.
- **Exclusion Criteria:** Non-peer-reviewed publications (posters, extended abstracts, demos, editorials), duplicate works, studies not addressing the defined research questions, papers lacking empirical or theoretical contributions, and non-English publications.

### 3.3 Screening process

After the first database search, a multistage screening procedure was applied. Records that were irrelevant, off-topic, or duplicates were eliminated through title and abstract screening. A complete text review of the remaining articles confirmed their relevance and quality was confirmed. Throughout the review, the inclusion and exclusion criteria were consistently applied and without exception.

### 3.4 PRISMA 2020 flow diagram

**Figure 1:**
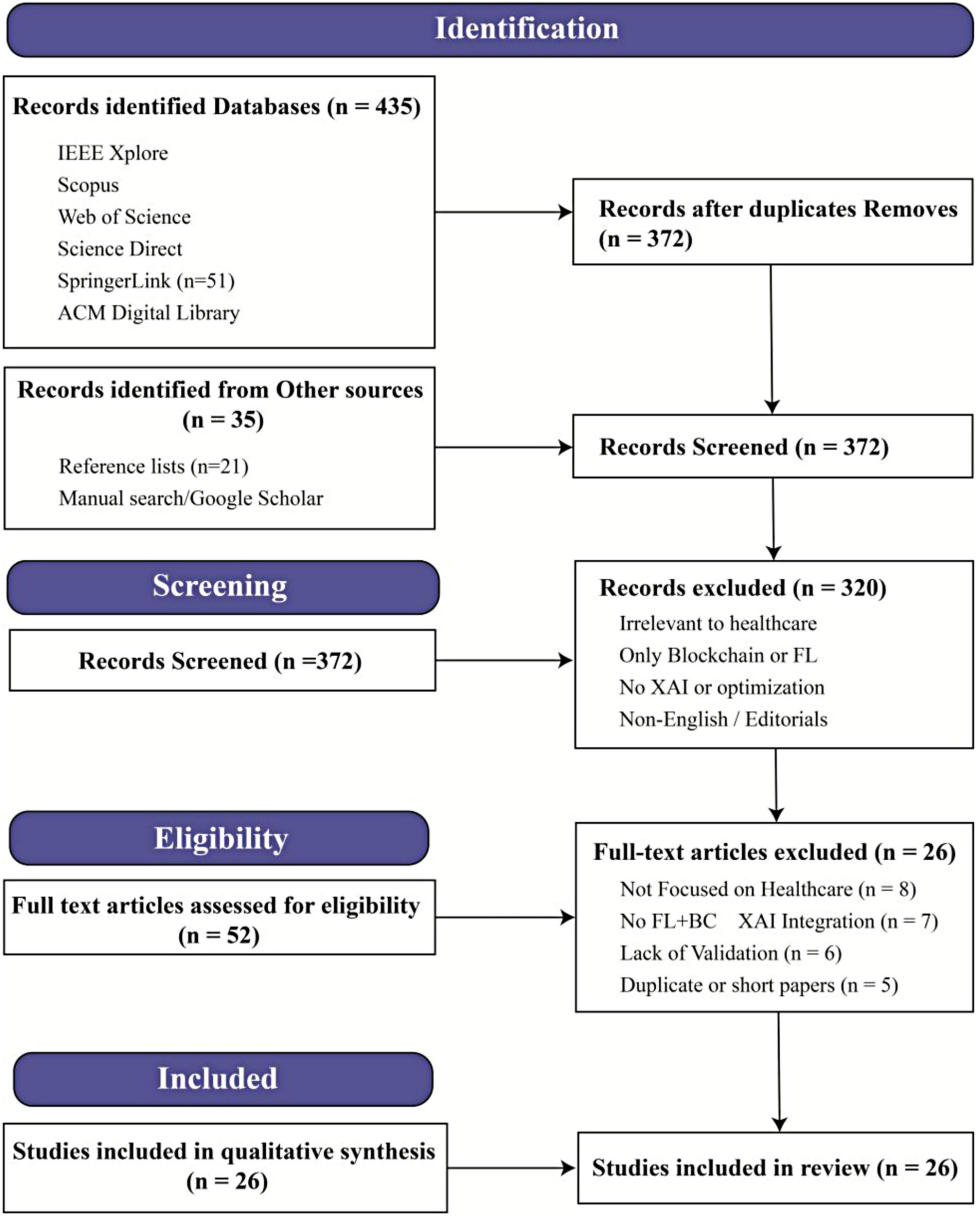
PRISMA 2020 flow diagram for systematic literature review.

Figure 1 presents the PRISMA 2020 flow diagram illustrating the four-stage systematic screening process applied in this review to select the studies.

**Figure 2:**
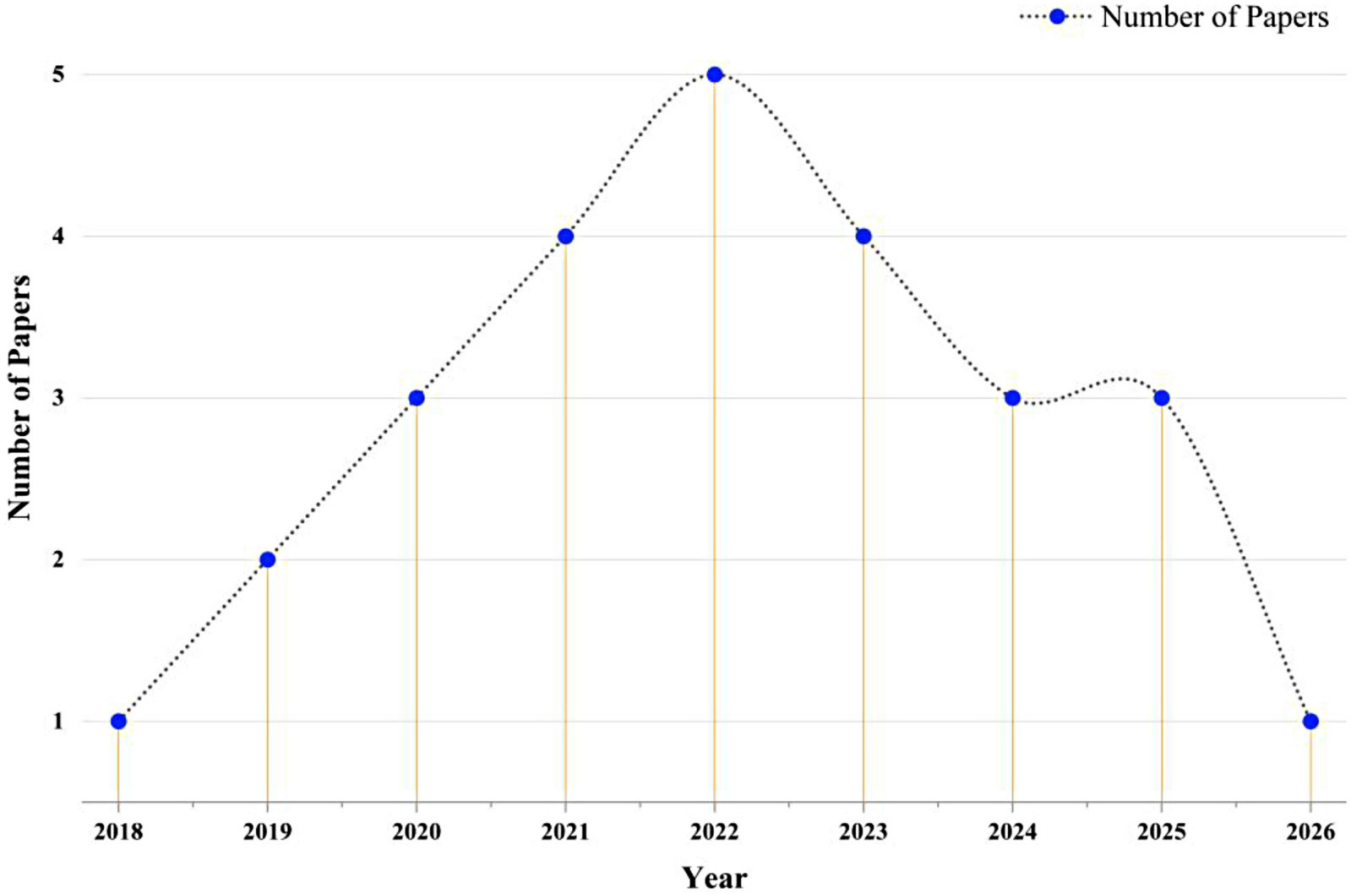
Annual distribution of reviewed studies (2018–2026), illustrating a peak in research activity in 2022 and sustained interest until 2026.

The study selection counts recorded above are consistent with the systematic screening procedure described in Section 3. A total of 470 records were identified (435 from databases and 35 from manual sources). After removing 98 duplicates, 372 records were screened. Based on the title and abstract evaluation, 320 records were excluded. Of the 52 full-text articles assessed for eligibility, 26 were excluded (8 not focused on healthcare, 7 lacking FL/BC/XAI integration, 6 lacking validations, and 5 duplicates or short papers). A final set of 26 studies was included in the qualitative synthesis across five thematic categories: Blockchain-based EHR frameworks, Federated Learning in healthcare, Explainable AI in healthcare, Integrated FL + Blockchain + XAI frameworks, and Incremental Optimization in healthcare AI. Figure 2 illustrates the annual distribution of the reviewed studies, revealing a steady growth in research activity from 2018 and a peak of five publications in 2022.

### 3.5 Quality assessment

The methodological quality of all 26 included studies was systematically evaluated using the Critical Appraisal Skills Programme (CASP) Qualitative Checklist, a widely validated and internationally recognized instrument designed to appraise the rigor, credibility, and relevance of qualitative and mixed-methods research studies. The CASP Qualitative version was selected in preference to alternative appraisal tools such as AMSTAR-2, which is designed for meta-analyses, or the Newcastle-Ottawa Scale, which targets cohort and case-control studies because the heterogeneous and integrative nature of this review encompasses a broad spectrum of research designs, including empirical system evaluations, architectural proposals, and secondary literature reviews.

The checklist comprises ten appraisal criteria organized across three thematic domains, as summarized in Table 2. Each criterion was scored on a three-point scale: “Yes” (criterion clearly met, one point), “Can’t Tell” (insufficient information, zero points), or “No” (criterion not met, zero points), yielding a maximum composite score of 10 per study.

**Table 2:** CASP qualitative checklist criteria.

Code	CASP criterion	Domain	Scoring scale
C1	Clear statement of research aims	Internal Validity	Yes/CT/No
C2	Appropriateness of qualitative methodology	Internal Validity	Yes/CT/No
C3	Rigor of research design	Internal Validity	Yes/CT/No
C4	Adequacy of data collection strategy	Research Process	Yes/CT/No
C5	Rigorous data analysis procedures	Research Process	Yes/CT/No
C6	Researcher role/reflexivity	Research Process	Yes/CT/No
C7	Consideration of ethical issues	Research Process	Yes/CT/No
C8	Credibility and clarity of findings	Relevance & Value	Yes/CT/No
C9	Value and relevance of the research	Relevance & Value	Yes/CT/No
C10	Contribution to existing knowledge	Relevance & Value	Yes/CT/No

**Figure 3:**
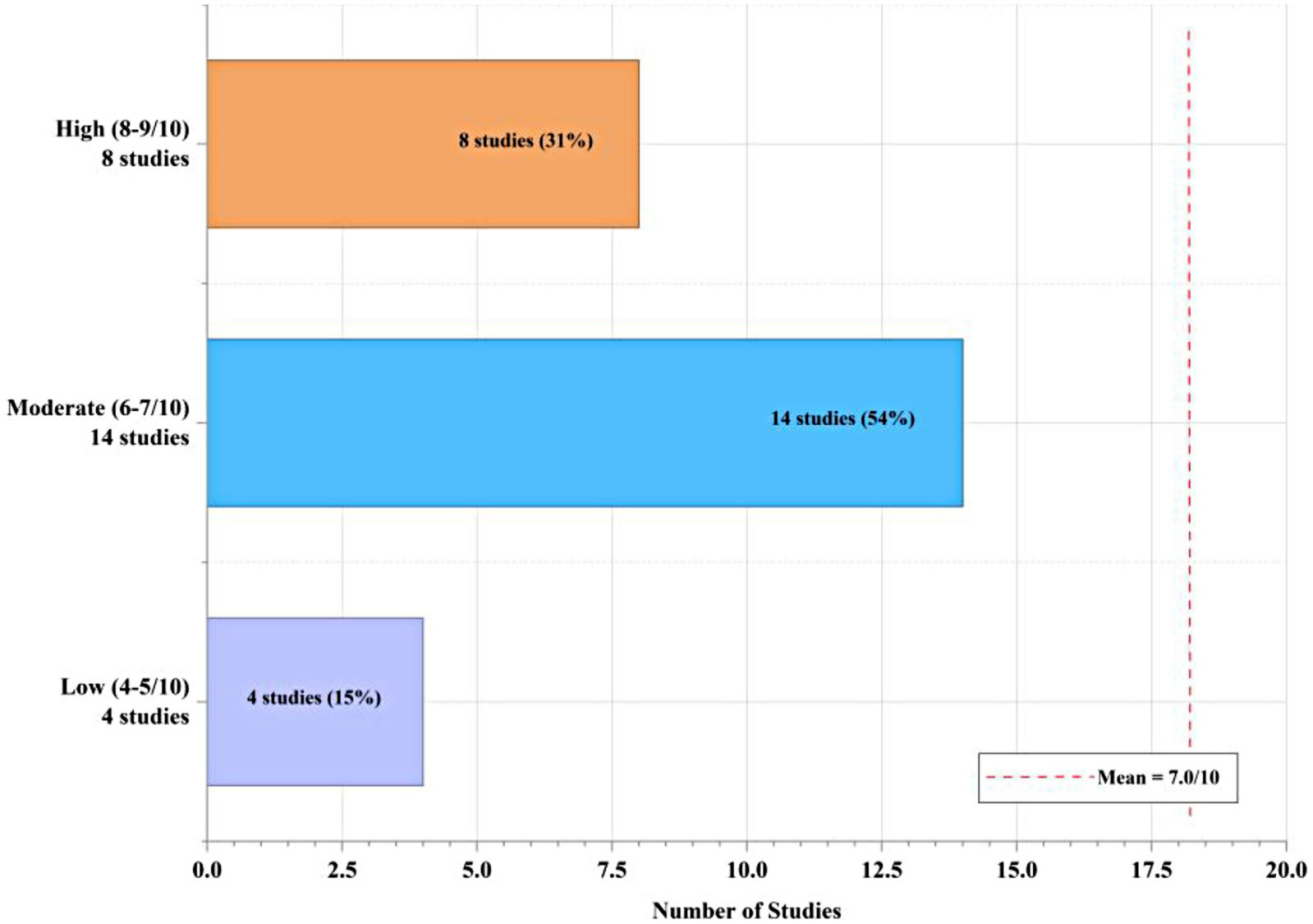
Distribution of the CASP quality scores.

The appraisal process was performed separately by two independent reviewers for all 26 included articles, in which both reviewers scored all included studies without knowing the rating provided by the other reviewer, thereby reducing evaluator bias. Inter-reliability was ensured by using a standardized scoring technique for all studies included. If differences occurred in the scoring of the included studies, consensus was achieved through deliberation. In situations where no agreement could be reached, a third independent reviewer was contacted to make the final decision. Quality appraisal in this review served as a tool for transparency and interpretation rather than being a means of filtering out studies based on their CASP scores. All 26 studies were considered irrespective of their scores, and quality was only used to inform the extent of weight given to a particular study finding when synthesizing evidence; that is, studies that showed weak methodological rigor would be treated accordingly. This approach is in line with practices in integrative and scoping reviews, where evidence completeness becomes important, together with critical awareness of study limitations. Figure 3 shows the distribution of CASP quality score in all 26 included studies, in which more studies had moderate quality.

The following characteristics were observed throughout the scoring process. Virtually all selected articles met criteria C1, C2, C8, and C9, showing consistent research framing and methodology reporting. Criterion C6, related to researcher role and reflexivity, was not met in the case of technology-engineering research. This is perfectly normal according to the rules of scientific writing in applied computing research field. Criterion C7, on ethics, got “Can't Tell” ratings in most of the analyzed studies. The analysis of health data shows that researchers care about ethical issues; however, there are few instances in which researchers have explicitly mentioned ethical approval in engineering-related journals. A maximum score of 8/10 was assigned to studies reporting empirical results using benchmarked data and quantitative metrics. Surveys and conference proceedings with little evidence of validation got 4–5/10 scores. The average CASP score for all 26 studies included in this review was 7.0 out of 10, which shows acceptable methodological quality, sufficient for conducting a systematic review of the literature.

## 4 Scope of this work

This survey examines how Federated Learning, blockchain, Explainable AI, and incremental optimization can be integrated to improve data security, privacy, trust, and operational effectiveness in next-generation healthcare systems. Table 3 summarizes the eight dimensions.

**Table 3:** Scope dimensions covered.

Dimension	Coverage in this survey
Holistic framework integration	Integration of FL, Blockchain, XAI, and Incremental Optimization into a coherent unified architecture for healthcare data management
Privacy preservation	Differential Privacy, Homomorphic Encryption, and Secure Multi-Party Computation for privacy-preserving collaborative learning
Blockchain trust & integrity	Secure data sharing, immutable data storage, and smart contract-based access control
Explainability in AI	SHAP, LIME, and Attention Mechanisms for clinical transparency and trust
Incremental & adaptive learning	Online learning, continual learning for preventing catastrophic forgetting, and adaptive optimizers for dynamic healthcare datasets
Application domains	Disease diagnosis, medical imaging, remote patient monitoring, drug discovery, and personalised medicine
Edge & cloud integration	Edge computing for low-latency local processing; cloud computing for large-scale data analytics
Future research directions	Lightweight blockchain frameworks, explainable federated learning, energy-efficient optimization, 5G/6G and IoT integration

## 5 Literature review

**Figure 4:**
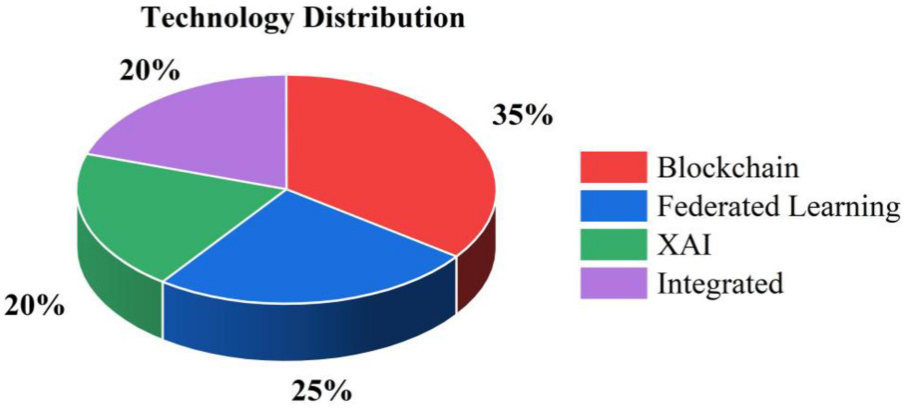
Proportional distribution of reviewed studies across four primary technology domains.

Figure 4 summarizes the proportional distribution of the 26 reviewed studies across the four technology domains, with blockchain accounting for the largest share at 35%.

### 5.1 Blockchain-Based EHR frameworks

Substantial research has been conducted on the use of blockchain technologies to secure electronic health records. Al-Khasawneh et al. (13) put forward a comprehensive blockchain framework consisting of five components, including smart contracts, encryption, privacy key management and integration with healthcare IT for secure and confidential use of patient data.

HealthBlock, introduced by Zaabar et al. (14), uses OrbitDB, which runs on IPFS and a Hyperledger Fabric blockchain, to store electronic health record (EHR) data. According to the Hyperledger Caliper performance benchmarks, it exhibits good throughput and latency behavior. Mondal et al. (15) developed a multi-signature blockchain architecture that solves the problem of data ownership through a private channel framework that allows patients to own their medical records. The joint results show that a blockchain-only framework can achieve data immutability and access control. Despite their utility, they all have the same drawback: none of them integrate machine learning or AI components.

### 5.2 Blockchain-Based EHR management with predictive features

Ismail and Materwala (16) presented BlockHR, a system constructed on the blockchain that enables patients to contribute lifestyle and medical data. This includes predicting chronic diseases. The framework strikes a balance between secure record management and the predictive capabilities. Gupta et al. (17) introduced an HSPBCI model, which combines blockchain with cloud-IoT services. The authors claimed that the new model encryption and decryption speed-up performance is over five times faster than the existing O(n^2^) one with O(n) complexity. In addition, the transaction processing time was between 0.15 and 0.44 ns. Jakhar et al. (18) presentation showcased a consensus-driven access-control framework that is privacy-preserving in nature. The mechanism implemented on Hyperledger Fabric manages access control in a multi-stakeholder environment. This architecture enhances security, compliance, and flexibility. These studies expand the literature by incorporating predictive or performance optimization dimensions into security. However, there is an important gap regarding the lack of federated learning and explanation.

### 5.3 Secure EHR sharing and storage architectures

Kumar et al. (19) designed a protocol that makes use of the Ethereum blockchain and IPFS that give the patients the full ownership of their EHR. The Blockchain Security Framework (BSF-EHR) was presented by Abunadi and Kumar (20) to provide an efficient and secure way of accessing EHR for physicians, patients, and insurance agents. The simulation results of BSF-EHR proved its ability to protect the EHR. Abbas et al. (21) developed Blockchain-Assisted Secure Data Management Framework (BSDMF) for IoMT environments, achieving 97.2% accuracy and 97.9% precision with 98.3% average trust value and 15.6% improvement in latency ratio. Ultimately, BlockHR provides 20× faster data retrieval than traditional client-server methods, but its execution time to write 2.6× longer, as evaluated by Ismail et al. (22). Collectively, these studies establish a strong empirical foundation for the use of blockchain in healthcare security. However, across all reviewed works in this category, the absence of explainability mechanisms and adaptive learning components remains a consistent limitation, underscoring the need for the integrated framework proposed in this study.

### 5.4 Federated learning in healthcare

Several researchers have shown that Federated Learning works well in healthcare applications. Alam and Gupta (24) conducted a comprehensive survey of federated learning over a wide range of applications, platforms and real systems. It involves the creation of private models without sharing raw data. The authors also provided IoT-based distributed implementations for secure healthcare deployment. Elayan et al. (25) developed a deep Federated Learning framework for applications using IoT devices for healthcare monitoring. Their proposed method achieved a 97% AUC in detecting skin diseases while preserving patient privacy and reducing the operational costs. Singh et al. (26) proposed an architecture for training a model in a decentralized fashion without opening up data fed from smart healthcare and smart cities using blockchain and federated learning. Federated learning was integrated with deep learning for IoT-based healthcare systems for enhanced privacy using homomorphic encryption and dropout-tolerant mechanisms by Zhang et al. (27), who showed improved performance over the HAM10000 dataset. These studies collectively assert that FL overcomes privacy restrictions in distributed healthcare. However, the lack of explainability mechanisms and absence of evaluation benchmarks at each federated node represent research gaps.

### 5.5 Explainable AI in healthcare

The following studies focused on XAI in the healthcare domain. Some studies use centralized architectures, but they define the interpretability techniques SHAP and LIME, which are directly transferable to federated and distributed healthcare systems when used in combination with FL. Raza et al. (28) proposed an XAI-based module with federated transfer learning to obtain interpretable results for ECG classification which reaches an accuracy of 98.9% on the MIT-BIH dataset. They also proposed a communication cost reduction method to improve the privacy of the federated framework. Mienye and Jere (29) proposed a model for cardiac disease prediction by incorporating ensemble learning, Bayesian optimization, and SHAP explanations for performance and interpretability. The optimized XGBoost model produced high specificity and sensitivity for the Cleveland and Framingham data.

Uddin et al. (30) imposed a machine-learning framework that has the aspect of pre-processing and feature selection, data balancing, ensemble learning, and XAI modules; voting ensemble achieved 98.63% accuracy and 99.13% AUC-ROC when predicting cardiovascular disease; SHAP and LIME provided explanations for each prediction with clinical reasoning. Similarly, Khawla et al. (31) suggested an explainable ensemble framework that uses data balancing with SMOTE. Here, CatBoost achieved the highest accuracy of 99.44% and a strong ROC-AUC, with SHAP and LIME having clinically meaningful interpretability. Despite using a centralized architecture, the SHAP-based feature attribution of both Uddin et al. and Khawla et al. can provide building blocks for interpretability that may be effectively transferred to federated healthcare settings, which has become an important challenge for clinical transparency. According to the collective of the above studies, explainability and accuracy are complementary and not competing objectives in healthcare AI, which is a principle at the heart of the integrated framework advocated in this survey.

### 5.6 Integrated FL, blockchain, and XAI frameworks

A growing body of research has moved beyond single-technology approaches to propose integrated frameworks that combine Federated Learning, blockchain, and XAI for end-to-end secure and interpretable healthcare data management. Qammar et al. (34) conducted a systematic literature review on securing federated learning with blockchain, identifying immutable audit trails and smart-contract-based aggregation as the most effective mechanisms for hardening FL against model poisoning and Sybil attacks. Their analysis of 42 primary studies confirmed that blockchain integration reduces the single-point-of-failure risk in FL pipelines while introducing manageable computational overhead when permissioned chains are adopted.

Tariq et al. (36) proposed a blockchain-based secure data sharing framework employing attribute-based encryption combined with on-chain access logs, validated against HIPAA compliance benchmarks—one of the few frameworks in the literature to explicitly address regulatory alignment alongside technical performance. Wang et al. (38) synthesized findings across 37 studies and identified gradient compression and asynchronous aggregation as the most promising techniques for reducing communication overhead. Ngoupayou et al. (39) demonstrated a 34% reduction in privacy leakage compared to standard FL aggregation using zero-knowledge proofs and blockchain-based update verification.

Hiwale et al. (45) reviewed 31 studies on remote patient monitoring, teleconsultation, and medical image sharing, identifying latency as the most critical unresolved barrier for real-time telemedicine applications. Das et al. (41) demonstrated that privacy and interpretability can be jointly optimized in a privacy-aware, deep learning system, challenging the assumption that these objectives are inherently in tension. Collectively, these integrated studies confirm that combined FL, Blockchain, and XAI architectures are technically feasible and represent the current frontier of secure, transparent, and regulatory-compliant healthcare AI.

### 5.7 Incremental optimization in healthcare AI

Incremental and adaptive learning methods address the fundamental limitation of static machine learning models in healthcare: the inability to adapt to evolving patient data distributions without complete retraining. Schuster et al. (42) demonstrated that incremental process discovery maintained model accuracy within 3% of full-batch retraining while reducing the computational cost by up to 68%, evaluated across three hospital datasets. This finding establishes a strong empirical case for incremental approaches in resource-constrained clinical settings.

Several continual learning and adaptive optimization algorithms can improve the effectiveness of incremental healthcare intelligence. Elastic Weight Consolidation (EWC), Replay-Based Continual Learning, Synaptic Intelligence, and Online Sequential Learning are commonly used to reduce catastrophic forgetting during continuous model updates. In addition, adaptive optimization strategies, such as Adam, RMSProp, Adaptive Gradient Descent, and Federated Averaging with dynamic learning rate adjustment, improve convergence stability and reduce communication overhead in federated healthcare environments. These approaches enable healthcare models to adapt efficiently to evolving patient data distributions, while maintaining predictive reliability and computational efficiency.

Bhanja et al. (43) highlighted adaptive ensemble methods and concept drift detectors as particularly effective for EHR anomaly detection, as patient population distributions shift continuously with seasonal disease patterns and evolving clinical practice. This study identified catastrophic forgetting as the primary technical barrier to deploying incremental models in federated healthcare settings. Eden et al. (47) identified continuous model adaptation as a key governance challenge in federated learning, noting that static consent frameworks are ill-suited for models that are updated dynamically over time. Taken together, these studies establish that Incremental Optimization is not merely a performance enhancement but a necessity for deploying FL-based healthcare systems in production environments where data distributions shift and regulatory requirements evolve.

The reviewed studies demonstrate substantial progress in secure healthcare data management using Federated Learning, blockchain, Explainable AI, and incremental optimization. However, most existing studies primarily focus on individual technologies or limited hybrid integrations rather than a unified framework. Several studies emphasize privacy preservation and decentralized learning, whereas fewer investigations simultaneously address interpretability, adaptive optimization, and real-time deployment. In addition, many frameworks are validated only on limited datasets or simulated environments, restricting their practical applicability in large-scale healthcare systems. Therefore, a stronger integration of secure decentralized learning, explainable decision-making, adaptive optimization, and scalable blockchain architectures remains an important research direction for future healthcare intelligence systems.

### 5.8 Comparative summary of reviewed literature

Table 4 presents a comparative summary of the reviewed studies, categorizing each by its primary technology domain, methodology, dataset, and key findings**.**

**Table 4:** Comparative summary of reviewed studies.

Author/Year	Technology	Privacy method	Key contribution	Limitation
Al-Khasawneh et al. (2024) (13)	Blockchain + Smart Contracts	Encryption + Key Mgmt	Secure EHR framework with 5-component architecture	No FL or XAI; centralized AI risk
Zaabar et al. (2021) (14)	Blockchain (Hyperledger) + IPFS	Access Control	OrbitDB-based decentralized EHR with Caliper evaluation	No FL; scalability concerns
Mondal et al. (2022) (15)	Blockchain Multi-signature	Data Decentralization	Patient-controlled EHR with multi-sig stamps	No privacy-preserving ML
Ismail and Materwala (2020) (16)	Blockchain (BlockHR)	Distributed Ledger	Chronic disease risk prediction + secure records	No FL or XAI
Gupta et al. (2025) (17)	Blockchain + Cloud IoT	Encryption (O(n))	5× faster encryption vs. existing models	No FL; no explainability
Jakhar et al. (2024) (18)	Blockchain (Hyperledger Fabric)	Access Control + Consensus	Privacy-preserving access control for EHR	No incremental learning
Kumar et al. (2022) (19)	Blockchain + IPFS + Ethereum	Homomorphic Encryption	Patient-owned EHR with smart contracts	No FL or XAI
Abunadi and Kumar (2021) (20)	Blockchain (BSF-EHR)	Encryption	Security framework for EHR with simulation results	No FL; no adaptive updates
Abbas et al., 2024 (21)	Blockchain + IoMT	Trust-Based Security	97.2% accuracy; 15.6% latency improvement	No XAI; no Incremental Opt.
Ismail et al. (2020) (22)	Blockchain (BlockHR)	Distributed Ledger	20× faster data retrieval vs. client-server	No FL; limited scalability
Alam and Gupta (2022) (24)	Federated Learning (FL)	Data decentralization	Comprehensive analysis of FL across platforms, applications, and IoT systems	Lack of experimental validation and performance metrics
Elayan et al. (2021) (25)	Deep Federated Learning (DFL) + IoT	Data locality (no sharing)	Proposed DFL framework for healthcare monitoring with 97% AUC in skin disease detection	Limited to specific application (skin disease), scalability not analyzed
Singh et al. (2022) (26)	FL + Blockchain + IoT	Blockchain security + decentralization	Secure architecture enabling privacy-preserving ML without data sharing	High computational complexity and integration overhead
Zhang et al. (2022) (27)	FL + Deep Learning + IoT	Homomorphic Encryption, cryptographic protection	Introduced dropout-tolerant FL with improved performance on HAM10000 dataset	Increased communication and computation cost
Raza et al. (2022) (28)	FL + XAI	Communication cost reduction + FL privacy	XAI-based framework achieving 98.9% accuracy with improved interpretability	Limited generalization beyond ECG datasets
Mienye and Jere (2024) (29)	Ensemble ML + XAI (SHAP)	Not explicitly defined	Integrated Bayesian optimization with XAI, achieving high specificity and sensitivity	No federated or decentralized learning considered
Uddin et al. (2026) (30)	Ensemble ML + XAI	SMOTE-based balancing	Voting ensemble achieved 98.63% accuracy and 99.13% AUC-ROC for CVD detection	Centralized model; lacks privacy-preserving mechanism
Khawla et al. (2026) (31)	Ensemble ML + XAI	SMOTE (data balancing)	CatBoost-based framework achieving up to 99.44% accuracy with strong interpretability	No FL or blockchain integration for data security
Integrated FL + BC + XAI Frameworks				
Qammar et al. (2023) (34)	FL + Blockchain	BC audit trails + smart contracts	SLR of 42 studies; BC aggregation identified as key defence against model poisoning	Survey only; no original empirical evaluation
Tariq et al. (2025) (36)	Blockchain + ABE	ABE + on-chain access logs	HIPAA-aligned secure data sharing; granular attribute-based access control	No FL integration; limited scalability analysis
Wang et al. (2026) (38)	FL + Blockchain (SLR)	Decentralised aggregation	SLR of 37 studies; gradient compression identified as key overhead reduction	Survey only; no new framework proposed
Ngoupayou et al. (2025) (39)	FL + BC + Zero-Knowledge Proofs	ZKP + BC verification	34% reduction in privacy leakage vs. standard FL; smart healthcare focus	Computational overhead of ZKP not fully evaluated
Hiwale et al. (2023) (45)	FL + BC (telemedicine SLR)	Privacy-preserving FL + BC	SLR of 31 studies; latency identified as critical barrier for real-time telemedicine	Survey only; real-time deployment not demonstrated
Das et al. (2026) (41)	FL + XAI + Differential Privacy	DP + SHAP explanations	Privacy-aware explainable FL preserving SHAP fidelity under DP constraints	Limited dataset evaluation; scalability not assessed
Incremental optimization in healthcare				
Schuster et al. (2024) (42)	Incremental Process Discovery	Online model updating	68% cost reduction vs. full retraining; accuracy within 3%; 3 hospital datasets	Limited to process mining; no FL or BC integration
Eden et al. (2025) (47)	FL Governance (scoping review)	Adaptive audit trails	SLR of 29 studies; continuous model adaptation identified as key governance challenge	Governance focus only; no technical framework proposed

## 6 Security aspects of healthcare data management

The system management of healthcare data should be accompanied by rigorous multilayer security. Patient healthcare data are sensitive (31). Healthcare records contain personally identifiable information, medical histories, diagnoses, and treatment information, the compromise of which can lead to serious ethical, legal, and financial issues. Within a framework that utilizes Federated Learning, Blockchain, XAI, and Incremental Optimization, security requirements go well beyond just protecting against the misuse of data but also encompass more than one layer in a distributed environment (32). Privacy and security play pivotal roles in every health system. There are core security requirements, which include confidentiality (the necessary protection from unauthorized access to patient data and model parameters), integrity (the necessary protection from unauthorized modification of data and model updates), availability (the guarantee of recipient access to the use of the service), authentication and authorization (the ability to verify the identity and enforce control of the participants) (33), and accountability and auditability (the ability to trace action or participants to demonstrate the compliance of necessary regulation, such as HIPAA and GDPR).

### 6.1 Security in federated learning

Federated Learning is susceptible to new attacks because of distributed training. Adversaries can reconstruct private patient data through gradient leakage. Membership inference attacks make it easy for adversaries to infer whether a particular patient's record was present in the training set (34). In a model poisoning attack, one or more attackers insert poisoned updates that corrupt the model training. Through backdoor attacks, hidden triggers are implanted to manipulate the output (35). First, a secure aggregation protocol using homomorphic encryption or multiparty computation can limit noise generation to the aggregator rather than all participants. This has the obvious advantage of better privacy preservation.

**Figure 5:**
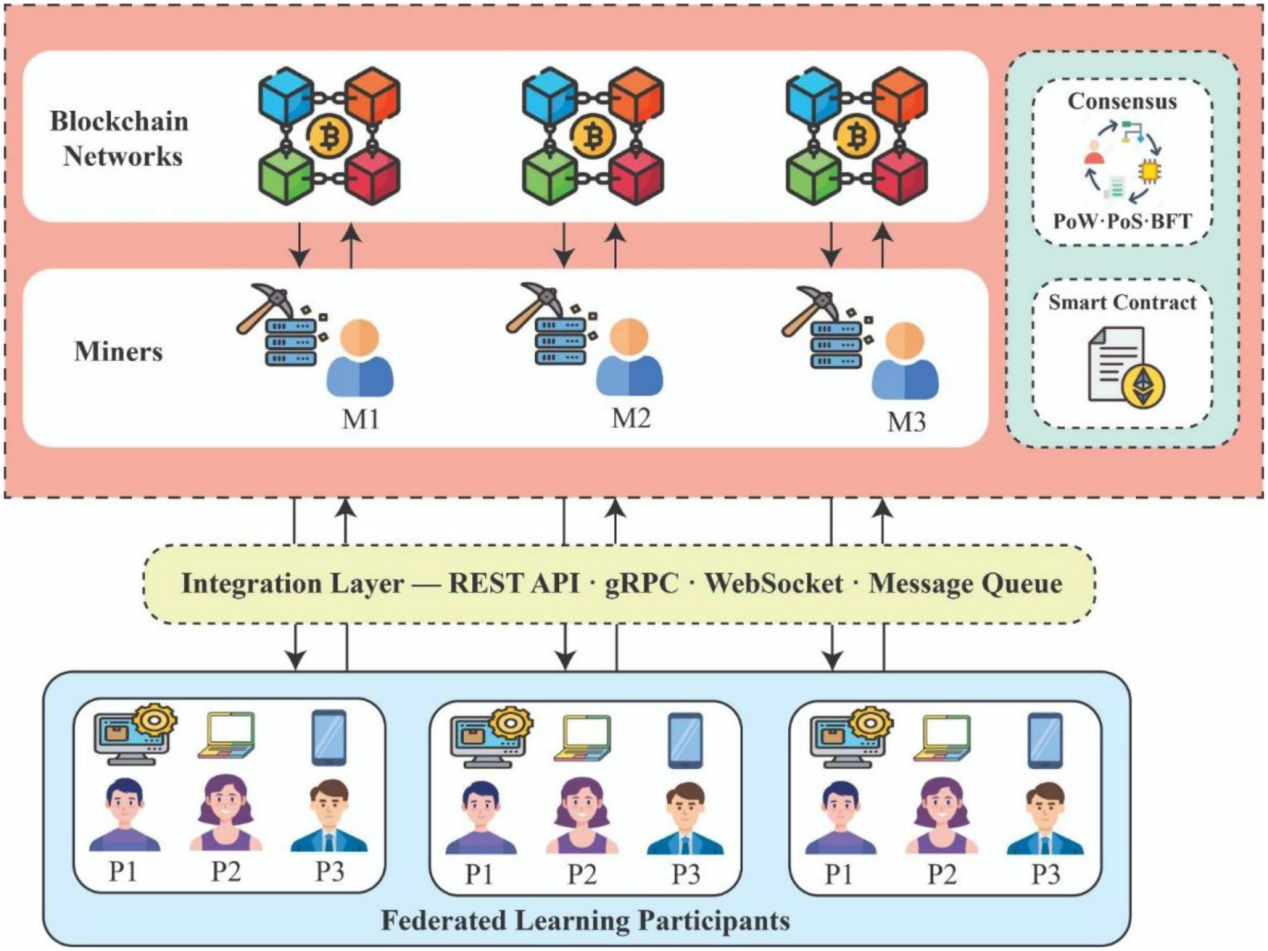
Integration of federated learning and blockchain for secure and decentralized healthcare model training.

Illustrated in (Figure 5) is an integrated blockchain–federated learning (FL) framework in which participants P1, P2, P3, etc. train local models with their private data without sharing the data. These participants send model updates via secure interfaces, such as REST API or gRPC. The integration layer links the FL participants and blockchain network to facilitate the secure transfer of model parameters. The blockchain layer is composed of multiple networks (M1, M2, and M3) and miners that validate transactions to ensure reliability (36). Consensus can be achieved using algorithms such as PoW, PoS, and BFT. Access control and confirmation of model updates are performed using smart contracts. Finally, a secure and decentralized structure is established through a design architecture based on federated learning to ensure data privacy and secure the blockchain. To inform the selection of an appropriate blockchain architecture for healthcare deployment, Table 5 presents a comparative analysis of public, private, consortium, and hybrid blockchains, evaluating their characteristics, advantages, limitations, and suitability for healthcare applications.

**Table 5:** Comparative analysis of blockchain types in healthcare.

Blockchain type	Characteristics	Advantages	Limitations	Healthcare suitability
Public Blockchain	Open decentralized network	High transparency and immutability	High latency and computational cost	Limited suitability
Private Blockchain	Controlled single organization	Fast transaction processing and privacy	Centralization risk	Suitable for hospitals
Consortium Blockchain	Shared among authorized organizations	Balanced security and scalability	Complex governance	Highly suitable
Hybrid Blockchain	Combination of public and private features	Flexible access control and interoperability	Complex implementation	Suitable for large healthcare ecosystems

### 6.2 Blockchain-Based security mechanisms

The blockchain, which is designed as a linked-block system, uses cryptographic hash functions. It is an indelible database system that creates a decentralized and tamper-proof record of transactions. Medical records can be managed using blockchains. Proof-of-Work (PoW), Proof-of-Stake (PoS), and Practical Byzantine Fault Tolerance (PBFT) are all consensus mechanisms that make a transaction valid. Healthcare applications prefer PBFT because of its lower latency and configuration in permissioned networks (37). With the help of smart contracts, access control and data-sharing policies will be automated. This helps reduce the chances of human error and unauthorized access. Scaling, computation costs, and storage overhead are challenges that remain for real-time healthcare deployment.

**Figure 6:**
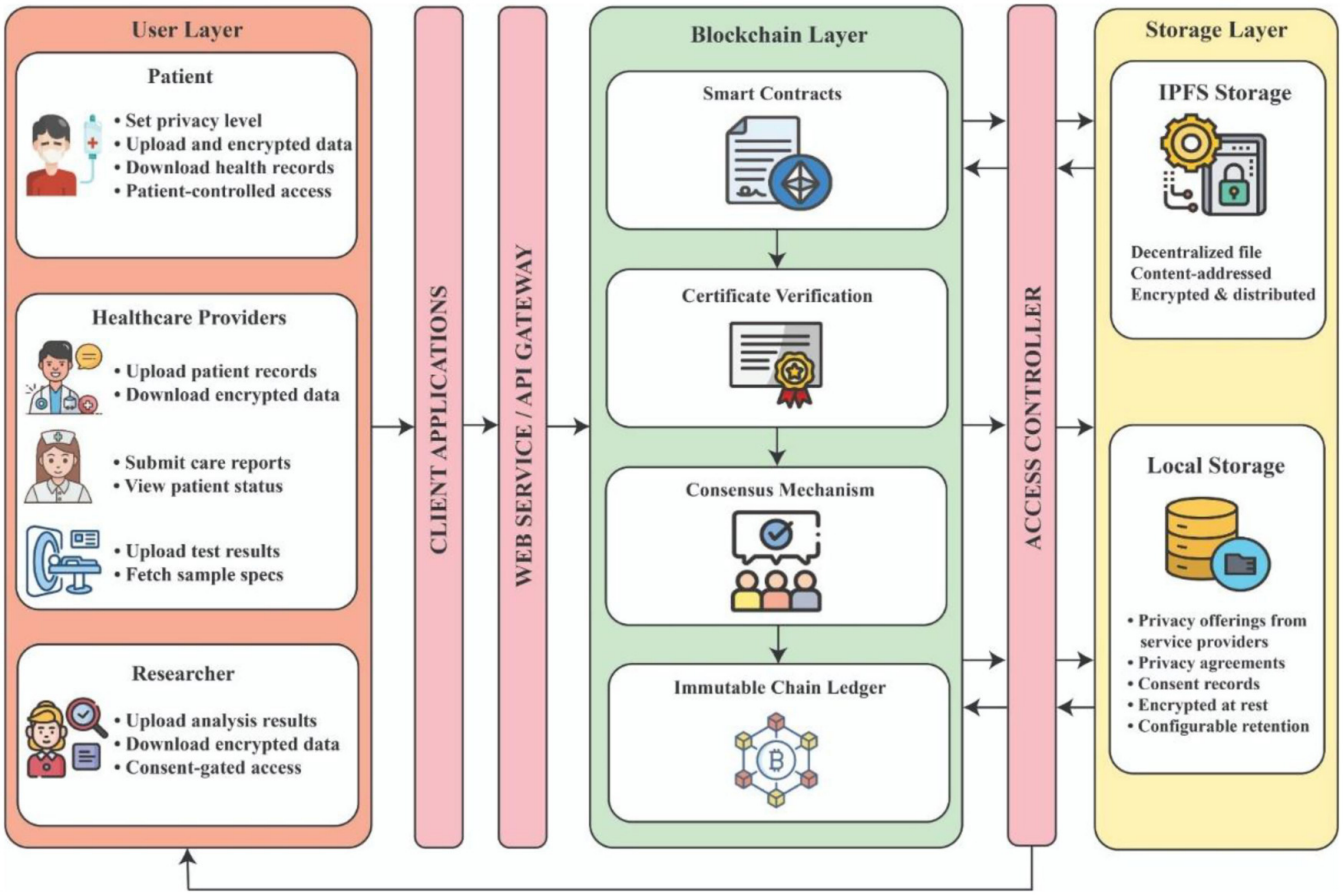
Blockchain-Based secure healthcare data management framework.

Figure 6 illustrates the architecture of the proposed secure blockchain-based healthcare data management system. It is composed of three layers: storage, blockchain, and users. Stakeholders, such as patients, healthcare providers, and researchers, use client applications to interface with the system. People can provide encrypted and access records, while providers and researchers can contribute to and retrieve protected health data. The Blockchain Layer ensures security and trust through smart contracts, certificate verification, and consensus mechanisms. Access restriction of smart contracts, certificate verification, authenticates users, and consensus ensures that they are fully validated before being posted to the immutable blockchain. The Storage Layer allows data storage in a decentralized manner, such as IPFS and local storage. Sensitive health data is off-chain encrypted. The blockchain saves the metadata and access logs. An access controller governs the secure data transmission over the layers. In general, the architecture is capable of safe, decentralized, and privacy-preserving management of healthcare data, which can ensure confidentiality, integrity, and access control while diminishing dependence on centralized systems.

From an operational standpoint, the three-tier design ensures a clear separation of concerns. For instance, the User Layer will take care of authenticating users and assigning access rights based on their roles via client applications. On the other hand, all transactions are verified through consensus algorithms while data sharing policies are enforced through smart contracts within the Blockchain Layer before committing the records to the blockchain ledger. Meanwhile, the storage of encrypted healthcare records occurs through the Storage Layer with IPFS and local databases; metadata and log data only are recorded in the blockchain.

### 6.3 Integration security: federated learning and blockchain

The fusion of FL and Blockchain offers a combined method for distributed security. By recording everything on the blockchain (immutable ledger), we assure the tamper resistance and traceability of all model updates (38). Thus, it drastically lowers the risk of a single point of failure. Incentive mechanisms that reward honest participants and penalize malicious ones are implemented using smart contracts (39). Therefore, smart contracts can support secure identity management. The main issue with this integration is the additional cost of blockchain transactions and FL operations in computing and communication.

**Figure 7:**
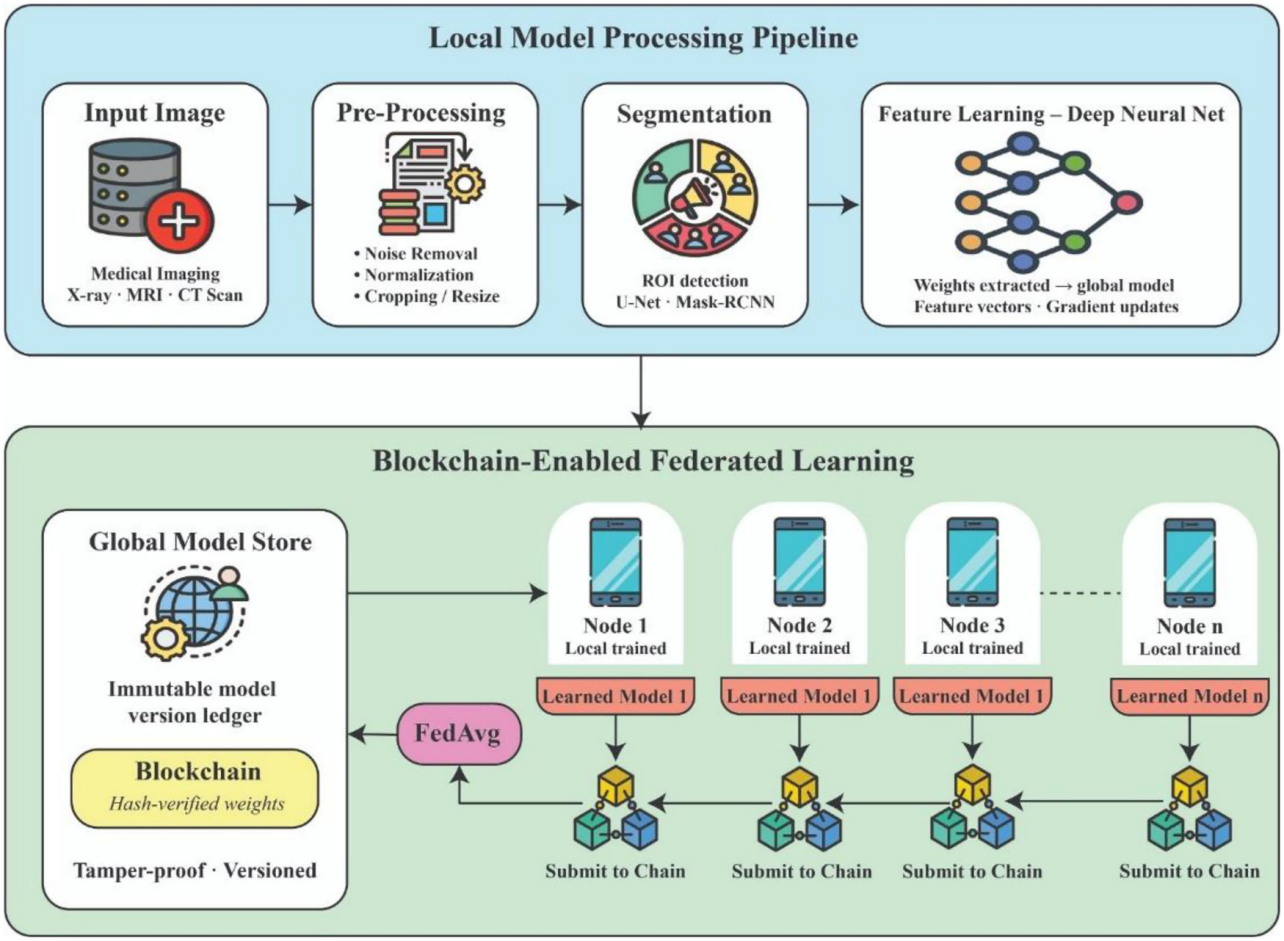
Federated learning–blockchain integration architecture.

Figure 7 illustrates the operational workflow of the blockchain-enabled federated healthcare learning. The architecture demonstrates how healthcare institutions can independently train local AI models using private patient datasets while preserving data confidentiality. Blockchain integration validates model updates using secure consensus mechanisms and stores immutable transaction records to ensure trust and transparency. The workflow also highlights decentralized aggregation, secure communication protocols, and collaborative model optimization, which collectively improve system scalability, privacy preservation, and resilience against centralized failure. Blockchain-enabled federated learning consists of two stages: local model processing and decentralized learning in healthcare. The input data from the doctor at the top was pre-processed and segmented. Deep learning models are then used to learn the features of the data. The distributed nodes at the bottom are Nodes 1 to n, which independently train their local models from their data. The raw data are not shared by these nodes to ensure data privacy.

The global model is stored in the blockchain layer, and node communication is secured. It preserves a permanent record of model updates and allows decentralized coordination without the need for a central server to manage the data. The architecture supports a secure, privacy-preserving, decentralized learning system, where federated learning allows for collaboration, and blockchain guarantees trustworthy, transparent systems and data integrity.

### 6.4 Security in explainable AI

Although XAI makes things more explainable, there are methods such as detailed feature attribution that can inadvertently reveal sensitive patient characteristics. Consequently, privacy inference has become a tangible risk (40). Adversaries may exploit explanation mechanisms to deduce the model architecture and identify exploitable weaknesses. Thus, privacy-aware explainability techniques that constrain explanation granularity while preserving interpretability are required. It is necessary to ensure robustness against adversarial manipulation of explanations and protect intermediate representations (41) using secure feature attribution methods.

**Figure 8:**
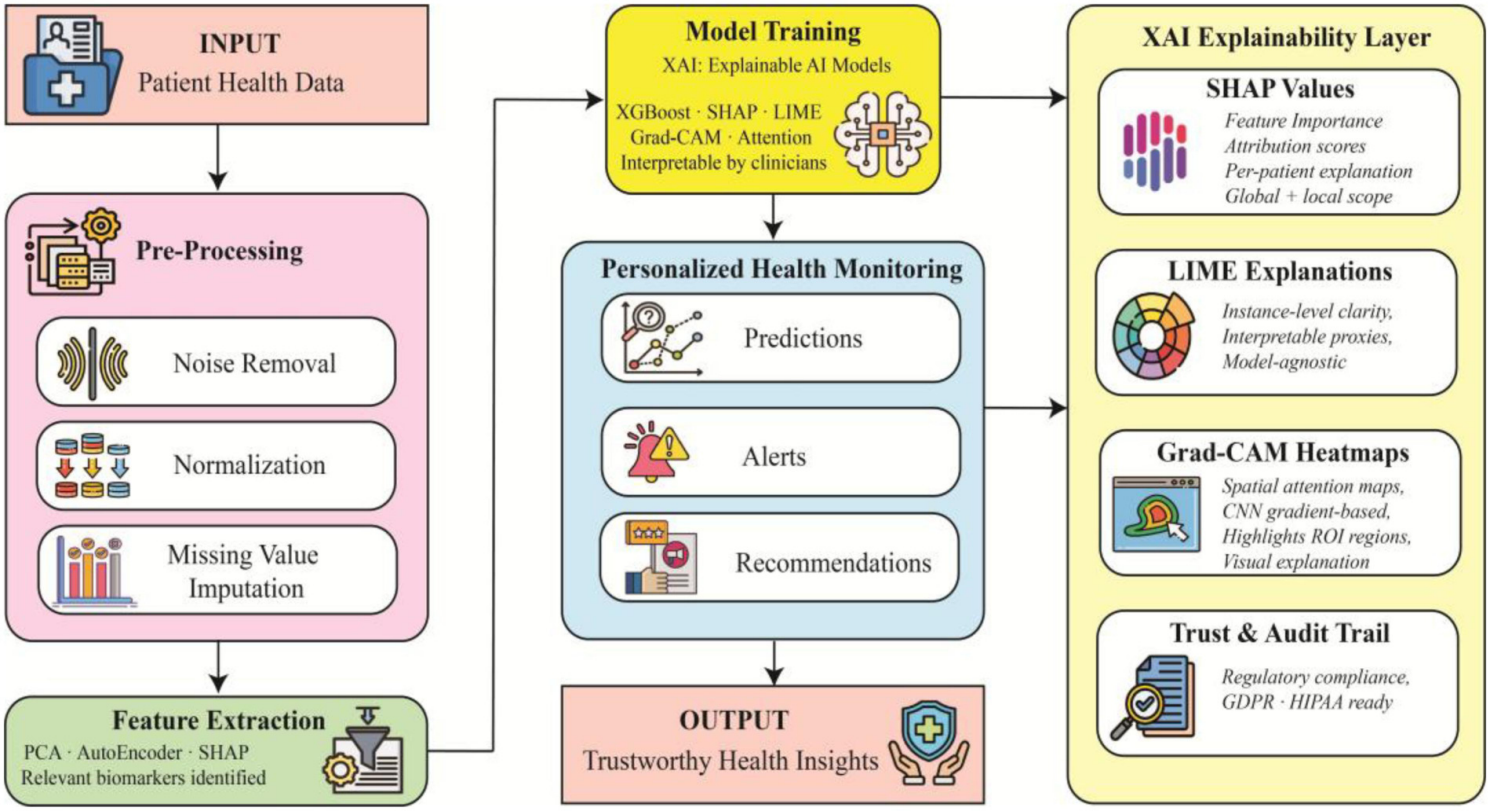
Explainable AI workflow with privacy risks.

The transformation of patient data into trustworthy clinical insights using the security-aware Explainable AI (XAI) pipeline of healthcare analytics is shown in Figure 8. It is now time to input the quality pre-processed health data of the patient into the software. The processing phase consists of noise removal, normalization, and missing value imputation, so that model reliability and inference are not hampered by incomplete and noisy medical data. After refinement, feature selection parameters are extracted, from which particular feature choices are made. The redundancies are reduced, which improves the efficiency and interpretability of the model. These are important traits for XAI systems whose explanations must be meaningful and clinically relevant. There is also an inherent trade-off between the granularity of explanations and the protection of privacy. There is, at each level from data preprocessing to feature selection, and finally to the model output, an information leakage surface. Feature-level attributions derived using methods like SHAP and LIME could unintentionally disclose patient-related sensitive information in the explanation results if the results themselves were shared without sufficient protection. The overall design takes into consideration a security perspective where differentially private approaches limit the granularity of explanations, while access to these more detailed explanation results is made available only to selected clinicians with suitable role-based permissions.

Human-centered evaluation plays a critical role in the adoption of Explainable AI in healthcare systems. Clinicians require transparent and interpretable AI predictions to ensure trustworthy clinical decision-making and patient safety. Explainability techniques such as SHAP, LIME, and attention visualization improve the understanding of model predictions by highlighting the important clinical features that contribute to diagnostic outcomes. In addition to predictive performance, factors such as usability, fairness, clinician trust, cognitive workload, and decision support reliability must also be evaluated. Human-centered evaluation frameworks can significantly improve the acceptance of AI-assisted healthcare systems by ensuring transparency, accountability, and ethical compliance in real-world clinical settings.

The processed information was used to train the XAI models. These models generate outputs for personalized health monitoring, which may include predictions, alerts, and recommendations. The output of XAI can explain itself, unlike black box models, which enables clinicians to understand the basis of the model's prediction. Ultimately, the system's output promotes healthy outcomes (42). Bringing together pre-processing, variable selection, and explainable modelling not only contributes to predictive accuracy but also assures transparency and reliability, which in turn ensures safe and secure AI deployment in healthcare decision-making.

### 6.5 Security in incremental optimization

Incremental model updating introduces a distinct set of security vulnerabilities that are frequently overlooked in static healthcare model deployments. The primary risks include model drift, in which gradual shifts in patient data distribution cause progressive degradation of model performance over time; adversarial data injection, in which a malicious actor deliberately introduces corrupted samples into the update stream to steer the model toward incorrect predictions; and noise accumulation, in which small errors in successive updates compound into significant performance loss across extended deployment cycles (43).

Several defensive mechanisms are essential in clinical practice to mitigate these risks. Regularization techniques, such as Elastic Weight Consolidation (EWC) and synaptic intelligence, constrain weight updates to prevent catastrophic forgetting while maintaining adaptability to new data distributions. Anomaly detection modules deployed at the update validation layer can identify and reject statistically aberrant updates before they are incorporated into the global model. Formal validation checkpoints at each update cycle ensure that the model performance on held-out clinical benchmarks does not fall below an acceptable threshold before the update is committed. Integrating blockchain-based update logging with incremental learning pipelines further strengthens security by creating an immutable audit trail of every model update, enabling retrospective forensic analysis when anomalous behavior is detected (42, 43).

### 6.6 Multi-Layer security architecture

An effective integrated security architecture comprises five complementary layers, as summarized in Table 6.

**Table 6:** Multi-Layer security architecture for integrated healthcare framework.

Security layer	Function	Key mechanisms
Data Layer	Encrypts all data at rest and in transit to protect patient privacy (44)	AES-256, TLS/SSL
Communication Layer	Protects data exchanged between distributed nodes using secure transport protocols	TLS 1.3, SSL
Model Layer	Applies Differential Privacy and secure aggregation to protect model parameters during FL	DP, HE, MPC
Blockchain Layer	Provides decentralized integrity, auditability, and access control via smart contracts (45)	Hyperledger, Ethereum
Application Layer	Enforces user authentication and XAI-based transparency for fair and accountable system operation	SHAP, RBAC, OAuth

**Figure 9:**
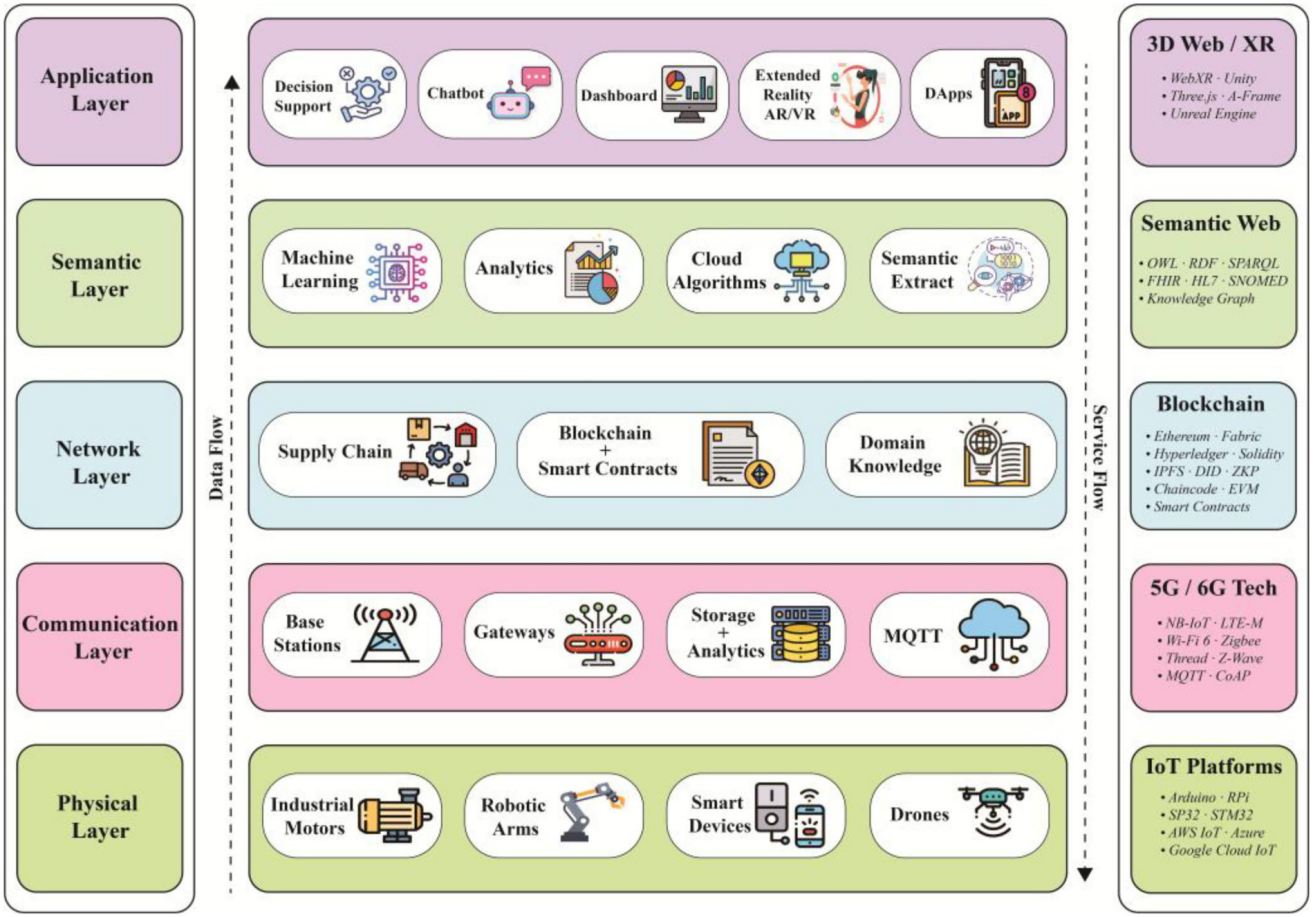
Multi-Layer security architecture.

The image illustrated in Figure 9 shows the architecture of a multilayered IoT, blockchain, and intelligent analytics system. The architecture is split into multiple layers, namely the Physical, Communication, Network, Semantic and Application layers. All smart sensors and gadgets send data through the Physical Layer, and the Communication Layer transmits this data to a gateway with the help of MQTT and 5G.

The network Layer uses blockchain with smart contracts and domain knowledge sources to allow secure decentralization. Using cloud, analytics, and ML techniques, the semantic layer processes data to derive meaningful insights. The Application Layer provides user-facing service dashboards, chatbots, and decision support tools.

As shown in the architecture outlined above, a secure, intelligent, and scalable system where data is processed in sequence from IoT-powered devices in the Physical Layer up to clinical decision support systems has been put into place, with each layer having its unique security and processing capabilities. By implementing a layered architecture model, separation of concerns can be achieved, meaning that there will not be any single layer possessing both the patient's data and the ability to process this information to ensure increased security and minimized risks. The integration of blockchain consensus validation technology and smart contracts for enforcing access control at the Network Layer ensures the creation of an immutable audit trail, helping organizations adhere to healthcare standards and guidelines regarding data security. As detailed in Table 6, each architectural layer is equipped with dedicated security mechanisms, collectively ensuring that data confidentiality, integrity, and availability are preserved throughout the entire processing pipeline.

The integration of Federated Learning, Blockchain, Explainable AI, and Incremental Optimization is technically feasible for modern healthcare infrastructures, particularly in distributed hospitals and IoMT environments. Federated Learning enables collaborative model training without sharing sensitive patient records, whereas blockchain ensures secure transaction validation, auditability, and decentralized trust management. Explainable AI improves clinician confidence by providing transparent predictions, whereas incremental optimization supports continuous model adaptation for dynamic healthcare data. However, practical deployment introduces several challenges, including communication overhead, interoperability issues, storage costs, blockchain latency, and computational resource limitations on edge devices. Hybrid edge-cloud architectures, lightweight blockchain consensus mechanisms, and optimized federated aggregation strategies can improve the deployment efficiency and scalability of real-time healthcare systems.

## 7 Results and discussion

### 7.1 Performance comparison of reviewed methods

The performance metrics from the most quantitatively assessed studies in this review are summarized in Table 7. The findings enable a comparative evaluation of the examined frameworks.

**Table 7:** Performance metrics of Key reviewed studies.

Author/Year	Method	Accuracy	Latency	Throughput	Key finding
Abbas et al. (2024) (21)	BC + IoMT	97.2%	11.2 ms	97.9% precision	Best accuracy among reviewed BC-only works
Gupta et al. (2025) (17)	BC + Cloud IoT	N/A	0.15–0.44 ns	5× faster than baseline	Fastest encryption/decryption reported
Ismail et al. (2020) (22)	BlockHR	N/A	20× faster reads	N/A	Best retrieval speed vs. client-server
Jakhar et al. (2024) (18)	Hyperledger Fabric	N/A	Low	High	Best compliance & access control
Zaabar et al. (2021) (14)	Hyperledger + IPFS	N/A	Moderate	High	Strong robustness via Caliper benchmark
Elayan et al. (2021) (25)	Deep FL + IoT	97% AUC	N/A	High	Best FL accuracy for skin disease; privacy-preserving IoT
Raza et al. (2022) (28)	FL + XAI (ECG)	98.9%	Low	High	Best FL + XAI accuracy (MIT-BIH); comm. cost reduced
Uddin et al. (2026) (30)	Ensemble ML + XAI (SHAP/LIME)	98.63%	N/A	High	98.63% acc, 99.13% AUC-ROC for CVD; SHAP + LIME interpretability
Khawla et al. (2026) (31)	CatBoost + XAI (SHAP/LIME)	99.44%	N/A	High	Highest XAI accuracy (99.44%); SMOTE balancing + CatBoost

**Figure 10:**
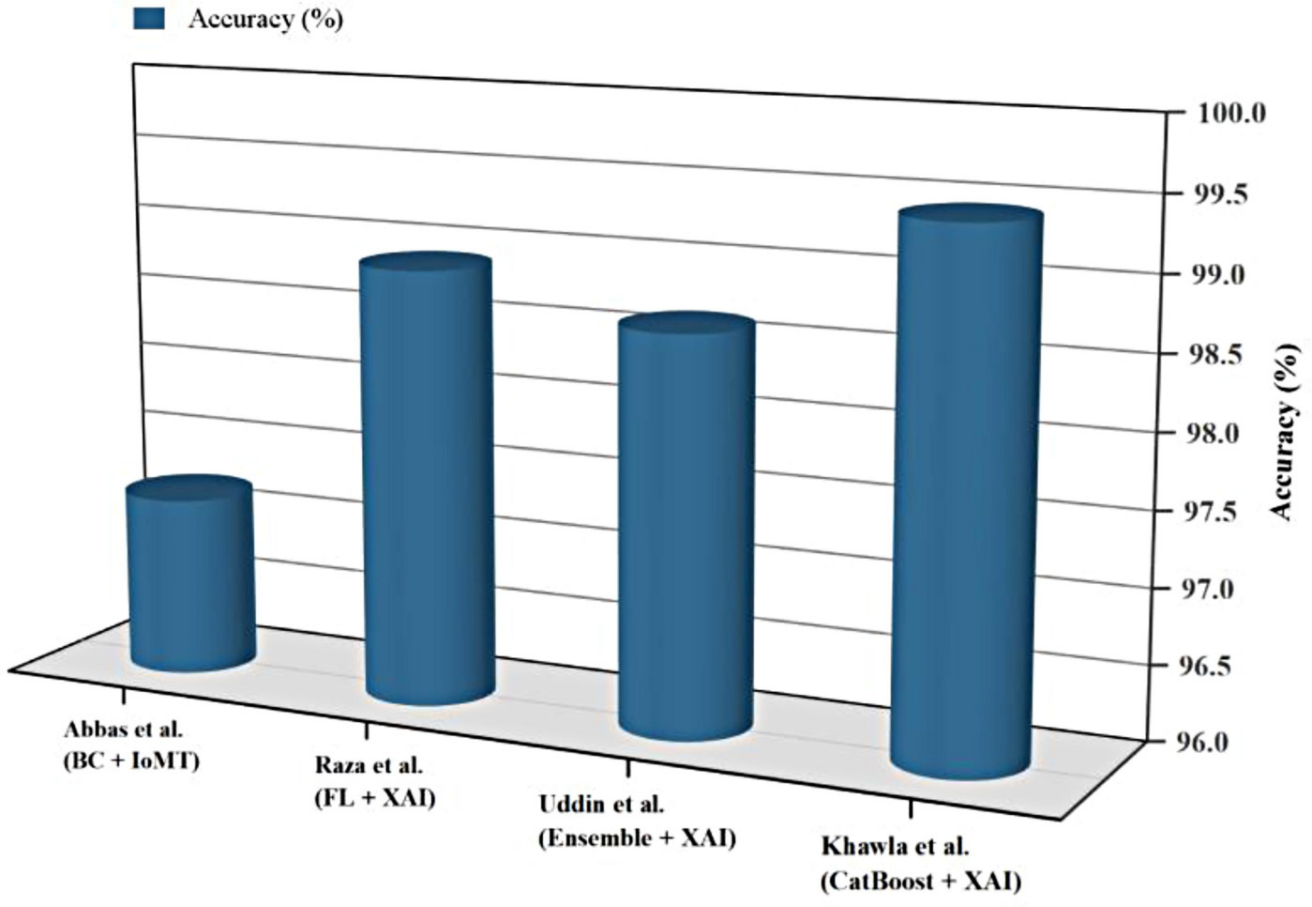
Accuracy comparison across the four studies reporting quantitative performance metrics.

Figure 10 presents a comparative view of the accuracy across the four studies reporting quantitative results, demonstrating that the XAI-integrated frameworks consistently achieved an accuracy above 98%.

Abbas et al. (21) reported the highest accuracy of 97.2% among the reviewed blockchain-based papers. The high accuracy is due to the trust-based IoMT security framework. According to the report by Gupta et al. (17) on encryption performance, the performance of O(n) shows a 5 times enhancement over the basic O(n^2^) models. As shown by Ismail et al. (22), blockchain-based retrieval can outperform traditional client-server architectures by 20 times, although with increased write latency, which is a crucial advantage for read-intensive clinical systems.

### 7.2 Challenge-to-Solution mapping

A key objective of this survey is to map the ten challenges identified in Section 2 to the solutions proposed in the reviewed literature, thereby revealing which challenges remain unresolved and warrant future research and mentioned in Table 8 (46).

**Table 8:** Challenges, existing solutions, and remaining research gaps in secure healthcare data management frameworks.

Challenge (Section 2)	Existing solutions from literature	Remaining research gap
Privacy leakage in FL	Differential Privacy, Secure Aggregation, HE	Optimal privacy-accuracy trade-off unsolved
Blockchain scalability	PBFT consensus, permissioned chains	Lightweight BC for real-time healthcare unresolved
Communication overhead	Gradient compression, model pruning	Low-bandwidth FL protocols needed
Data heterogeneity (non-IID)	Personalized FL, data augmentation	Robust convergence under extreme heterogeneity
Lack of interpretability	SHAP, LIME, Attention Mechanisms	Standardized XAI evaluation metrics missing
Integration complexity	Modular architectures	Unified open-source FL + BC + XAI frameworks lacking
Resource constraints	Lightweight models, edge computing	Energy-efficient protocols for IoMT unsolved
Security risks (poisoning, Sybil)	Anomaly detection, ZTA	Real-time adversarial detection in FL unresolved
Regulatory compliance	Smart contracts, audit logs	Automated GDPR/HIPAA compliance tools needed
Incremental learning challenges	Continual learning, EWC	Preventing catastrophic forgetting in FL + BC systems

### 7.3 Key trade-offs

- **Trade-offs Between Privacy and Accuracy:** The accuracy of models degrades with the application of differential privacy and homomorphic encryption. The optimal calibration of noise is an open issue that is currently under study.
- **Trade-off between Scalability and Security:** Security incurs costs. Enhanced security provided through more robust consensus protocols, such as Proof of Work, implies significantly lower transaction throughput. PBFT is one solution to the trade-off issue in permissioned blockchain networks in healthcare.
- **Trade-offs Between Transparency and Privacy:** Transparent fine-tuned XAI leads to a better understanding of models but risks privacy violations through sensitive attribute inference. Currently, techniques for offering explanations that preserve privacy are being studied.
- **Trade-offs Between Adaptability and Stability:** Adaptive continual learning via incremental learning results in model degradation (drift), which is mitigated partially through regularization techniques but not completely solved.

### 7.4 Advantages and Disadvantages of key Framework components

Table 9 presents advantages and disadvantages of key components in the integrated framework.

**Table 9:** Advantages and disadvantages of Key components in the integrated framework.

Component	Advantages	Disadvantages
Federated learning (FL)	Preserves data privacy; reduces breach risk; enables decentralized collaborative training	Vulnerable to gradient leakage and model poisoning; high comm. overhead; non-IID data issues
Blockchain technology (BC)	Immutability; transparency; auditability; eliminates central authority; smart contract access control	High computational cost; scalability issues; latency; complex integration
Explainable AI (XAI)	Enhances transparency; improves clinician trust; supports regulatory compliance	May expose sensitive info; risk of model reverse engineering; additional compute complexity
Incremental optimization	Continuous learning from new data; no full retraining needed; lowers compute cost over time	Model drift; catastrophic forgetting; noise accumulation; sensitive to adversarial inputs
Privacy-preserving techniques (DP, HE, MPC)	Strong privacy guarantees; secure collaborative learning; reduces data leakage	Trade-off between privacy and accuracy; high compute overhead; complex implementation
Integrated framework (FL + BC + XAI + Opt.)	End-to-end security; combines privacy, transparency, interpretability; real-time healthcare support	High system complexity; integration challenges; increased resource needs; deployment difficulty

**Figure 11:**
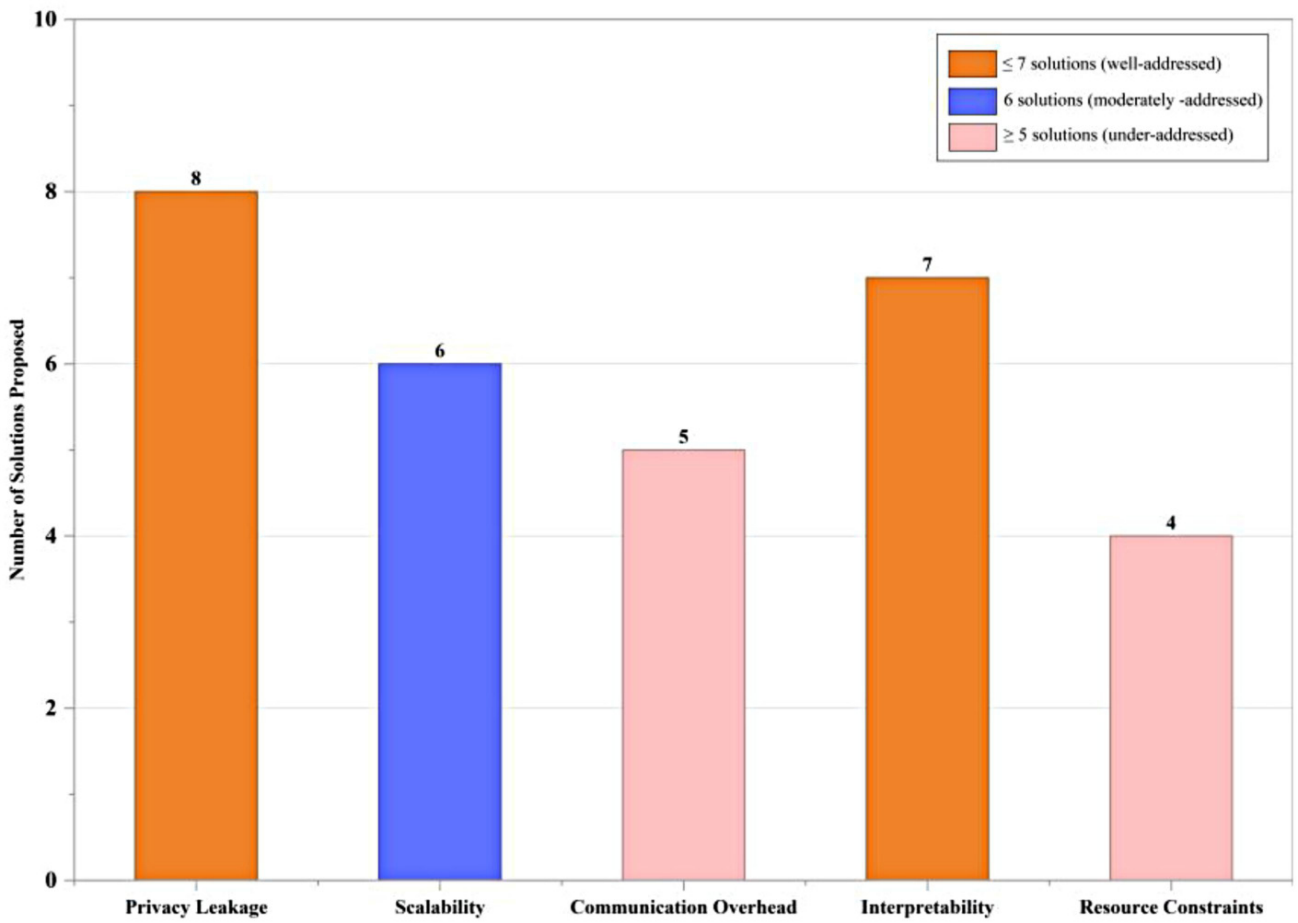
Number of proposed solutions identified for each research challenge.

Figure 11 visualizes the number of proposed solutions per challenge, indicating that Privacy Leakage and Interpretability are the most actively addressed, while Resource Constraints remain comparatively underserved.

### 7.5 Synthesis and implications

The integrated findings of the research analyses determined that the joint utilization of Federated Learning, blockchain, Explainable AI, and incremental optimization demonstrates the greatest effectiveness in the secure management of healthcare data, as no one technology on its own has enabled the secure management of data along with all security, privacy, and interpretability outcomes (47). Decentralized training ensures data privacy in federated systems. Blockchain technology offers access control while creating trust and immutability (48). Explainable Models enhance the essential transparency required for intelligent decision-making in the clinical decision-making process (49). The integrated framework is useful in extensive, large-scale, distributed healthcare systems, such as multi-hospital networks, IoMT-enabled remotely monitored environments, and telemedicine systems, which suffer from data heterogeneity and privacy challenges (50).

Many system designers may have in mind a minimum viable configuration with Federated Learning with secure aggregation, a permissioned blockchain (for example, PBFT-based) to validate updates to the model, and a lightweight XAI (51) component, such as SHAP or attention (52). Through Incremental Optimization, the model can continuously adapt and be effectively deployed. According to the review notes, the development of lightweight, scalable, and energy-efficient integrated frameworks, especially for resource-constrained IoMT environments, is the need of the hour (53). In addition, the challenge of achieving the right balance between privacy, accuracy, and explainability still exists. The results indicate that future work should examine a unified architecture to limit system complexity while maintaining adequate security for the real-world deployment of intelligent and trustworthy healthcare systems (23).

## 8 Applications of the integrated framework in healthcare

The federated learning-blockchain-explainable AI-incremental optimizer has a natural and meaningful application in a wide variety of clinical and operational healthcare settings. In the field of disease diagnosis and clinical decision support, FL facilitates hospitals to jointly train diagnostic models on distributed patient populations without centralizing sensitive records, while XAI components such as SHAP and LIME provide clinicians with interpretable rationales for model predictions, which is a prerequisite for adoption in high-stakes settings such as oncology, cardiology, and radiology. The study literature indicates that accuracy levels surpassing 98% have been achieved using this combination on the MIT-BIH and HAM10000 benchmark datasets (28, 31), thereby indicating readiness for clinical deployment for specific diagnoses. IoMT devices continuously generate physiological data from patients across different geographic locations in remote patient monitoring and telemedicine. Federated Learning helps in the local processing of data on edge devices, thereby safeguarding the privacy of users' data. Moreover, it allows for the incremental optimization of the model using this data in real time. In contrast, blockchain provides an auditable record of all data transfers and model optimizations. Overall, both these approaches directly address the latency and trust barriers, as highlighted by Hiwale et al. (45) and Elayan et al. (25).

The integrated framework has strong utility beyond diagnostics for Electronic Health Record management and secure multi-institutional data sharing. Smart contracts enabled by blockchain allow access control automation. Access to certain segments of data will only be provided to stakeholders such as patients, clinicians, insurers, and researchers. It will also have an immutable audit trail in accordance with the GDPR (11) and HIPAA (12). This model is relevant for multi-hospital network environments, which have occasionally been hampered in collaboration owing to data heterogeneity and institutional trust challenges. Incremental Optimization helps global models adapt over time as new institutions join or patient demographics shift. More importantly, the global model can adapt without the need to retrain the shared model.

The framework enables federated training among pharmaceutical research institutes while ensuring the privacy of proprietary compound data to support new drug discovery. The XAI methods will enhance the interpretability of the predicted DTIs, facilitating regulatory review and clinical translation. The applications, components, and benefits of the main technology categories are listed in Table 10.

**Table 10:** Application domains of the integrated FL + blockchain + XAI + incremental optimization framework.

Application domain	Primary technologies	Key benefit
Disease Diagnosis & Clinical Decision Support	FL + XAI (SHAP/LIME)	Accurate, interpretable predictions without data centralisation
Remote Patient Monitoring (IoMT)	FL + Incremental Optimization + Blockchain	Real-time adaptive monitoring with privacy and auditability
EHR Management & Secure Sharing	Blockchain + Smart Contracts	GDPR/HIPAA-compliant access control and tamper-proof audit trails
Multi-Hospital Collaborative Research	FL + Blockchain + Incremental Optimization	Privacy-preserving model training across institutional boundaries
Pharmaceutical Research & Drug Discovery	FL + XAI	Interpretable, privacy-safe drug-target interaction modelling
Telemedicine & Teleconsultation	FL + Blockchain + XAI	Secure, explainable remote clinical consultation support

## 9 Conclusion

In this study, we systematically and comprehensively examined the integrated application of Federated Learning, Blockchain Technology, Explainable Artificial Intelligence, and Incremental Optimization for secure healthcare data management. The results indicate that no single technology is up to the task for the security, privacy, and interpretability requirements of today's health systems. A framework that employs multiple technologies is required. Integrity and trust challenges are solved through blockchain, secure sharing mechanisms, and immutable and auditable data records. Federated Learning trains models while keeping patient data local to each institution. Explainable AI builds clinical trust through transparency and interpretable decision support for AI-based healthcare systems that lack interpretability. Through Incremental Optimization, models can dynamically adapt and continuously improve in evolving healthcare environments. Despite these advancements, challenges remain, including privacy vs. accuracy trade-offs, interoperability issues, resource constraints of edge devices, and security vulnerabilities such as model poisoning and adversarial attacks.

### 9.1 Future research directions

However, numerous critical areas require further exploration. Some recommendations for future research are as follows:

1. Scalable blockchain protocols to address latency and scalability issues in real-time health applications.
2. Less complex and less resource-intensive federated learning algorithms to minimize overhead costs and facilitate implementation in resource-limited environments.
3. A system to assess explainable artificial intelligence tools to foster interpretability and clinical trust.
4. Improvement in the stability and convergence of the federated learning algorithm by managing non-IID datasets and model drift.
5. Designing a common framework that combines federated learning, blockchain technology, XAI, and incremental optimization approaches.
